# Efficacy Assessment of Supramarginal Resection Versus Gross Total Resection in Glioblastoma: A Systematic Literature Review and Meta‐Analysis

**DOI:** 10.1002/brb3.71424

**Published:** 2026-04-16

**Authors:** Dipak Chaulagain, Volodymyr Smolanka, Andriy Smolanka, Kashif Qureshi, Kivanc Yangi, Oleg Devinyak, Bipin Chaurasia

**Affiliations:** ^1^ Neurosurgery Department, Regional Clinical Center of Neurosurgery and Neurology Uzhhorod National University Uzhhorod Ukraine; ^2^ Department of Neurosurgery Yale School of Medicine New Haven Connecticut USA; ^3^ Department of Neurosurgery Barrow Neurological Institute Phoenix Arizona USA; ^4^ Department of Pharmaceutical Sciences Uzhhorod National University Uzhhorod Ukraine; ^5^ Neurosurgery Department Neurosurgery Clinic Birgunj Nepal

**Keywords:** glioblastoma multiforme, supramarginal resection, gross total resection

## Abstract

**Background:**

The standard treatment for glioblastoma multiforme (GBM) typically involves a surgical resection followed by radiation therapy and chemotherapy. The extent of resection (EOR) plays a significant role in predicting the prognosis of GBM. The literature showed improved survival outcomes with greater removal of contrast‐enhancing tumor mass. We aim to compare the effects of supramarginal resection and gross total resection for the management of GBM.

**Methods:**

A systematic search was conducted from electronic databases (PubMed/Medline, Cochrane Library, and Google Scholar) from inception to February 10, 2024. All statistical analyses were conducted in Review Manager 5.4.1. Twelve studies meeting the inclusion criteria were selected. A random‐effects model was used when heterogeneity was observed to pool the studies, and the results were reported as hazard ratio (HR), odds ratio (OR), and standard mean difference (SMD), along with their respective 95% confidence intervals (CI). Primary outcomes were overall survival, Karnofsky Performance Status, and age; secondary outcomes included median progression‐free survival, mortality, and tumor recurrence.

**Results:**

Supramarginal resection was associated with improved overall survival compared with gross total resection (HR = 0.90, 95% CI 0.84–0.97; *p* = 0.005; *I* ^2^ = 96%), and increasing age was associated with decreased survival (HR = 1.03, 95% CI 1.00–1.05; *p* = 0.02; *I* ^2^ = 0%). No statistically significant association was observed between lower Karnofsky Performance Status and survival (HR = 0.77, 95% CI 0.53–1.12; *p* = 0.18; *I* ^2^ = 74%).

**Conclusion:**

Supramarginal resection of GBM yielded more favorable results than gross total resection, with minimal difference between adverse effects.

## Graphical abstract

Glioblastoma (GBM) carries a poor prognosis (median survival 15–18 months). Supramarginal resection (SMR) extends beyond contrast enhancement into T2/FLAIR to remove infiltrative tumor. In a meta‐analysis of 12 studies (6524 patients), SMR was associated with improved overall and progression‐free survival versus gross‐total resection (GTR) without higher adverse event rates, supporting SMR as a preferred extent of resection in selected patients.

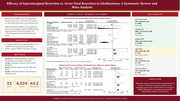

## 1 Introduction

Glioblastoma multiforme (GBM) is the most prevalent and aggressive form of glioma. Statistical evidence suggests a median survival time of 15–18 months, and the 5‐year survival remains below 10% (Wen et al. 2020). The standard treatment for GBM typically involves surgical resection followed by radiation therapy and chemotherapy (Wen et al. 2020; Marko et al. 2014). The extent of resection (EOR) plays a significant role in predicting the prognosis of GBM (Marko et al. 2014). The literature has shown improved survival outcomes with greater removal of contrast‐enhancing tumor mass (Marko et al. 2014).

GBM has characteristic radiological features: a nodule with contrast enhancement on T1‐weighted magnetic resonance imaging (MRI), surrounded by an irregularly shaped, widespread T2 hyperintense signal. This T2 signal is commonly believed to indicate infiltrated brain tissue (Stummer et al. 2008). Recent data suggest that complete resection provides greater benefits than partial removal or biopsy (Stummer et al. 2008; Chaichana et al. 2013). However, achieving complete resection is often challenging because gliomas infiltrate the surrounding brain tissue. Furthermore, the areas with lower cancer cell densities may not be visible on MRI scans, making it challenging for surgeons to accurately identify and remove all affected tissue during surgery (Baldock et al. 2013). Therefore, eliminating the tumor‐invaded brain tissue may not always be the optimal approach. As a result, the surgical strategy for brain gliomas is known as “maximal safe resection.” This approach aims to remove the tumor as much as possible without worsening the patient's condition. However, the precise extent of maximal safe resection lacks a standardized objective criterion, leading to different interpretations based on the surgeon's judgment (Marko et al. 2014; Karschnia et al. 2023).

Building on the concept of maximal safe resection, Duffau described and implemented supratotal resection (SpTR) in the setting of awake mapping, extending resection beyond the tumor margins to include adjacent infiltrated tissue, with favorable functional and oncologic outcomes reported in low‐grade gliomas (Duffau 2016). In subsequent literature, Yordanova et al. (2011) described this approach as supratotal resection (SpTR), which has also been referred to as supra‐complete, supramaximal, and supramarginal resection (SMR). In GBM, where true microscopic margins are not well defined, “supramaximal” may better capture the intent of safely maximizing tissue removal to improve overall survival without introducing new neurological deficits (Karschnia et al. 2023). The standards used to assess whether complete removal of all tumor tissue, known as gross‐total resection (GTR), has been achieved have historically relied on T1‐enhanced MRI following surgery. However, a recent investigation suggests that further extraction of areas displaying high signal intensity on T2‐FLAIR MRI can significantly enhance survival rates (Li et al. 2016).

In this meta‐analysis, we aim to compare functional outcomes in patients with GBM undergoing SpTR versus GTR.

## 2 Methods

### 2.1 Search Strategy and Databases

The systematic review was conducted in accordance with the Preferred Reporting Items for Systematic Reviews and Meta‐Analyses (Hutton et al. 2015). An electronic search was performed using PubMed/Medline, Cochrane Trial Register, and Google Scholar from inception through February 10, 2024. The following search string was used: (glioblastoma OR GBM OR high‐grade glioma OR glioma) AND (supramarginal resection OR supra total resection) AND (gross total resection OR complete resection). We additionally searched the referenced articles of previously published meta‐analyses, cohort studies, and review articles to identify any relevant studies.

### 2.2 Study Selection Criteria

Studies were selected if they followed our PICOS: P (Patients): Patients with Glioblastoma multiforme, glioma, or high‐grade glioma; I (Intervention): supratotal/supramarginal resection of the tumor; C (Control): gross total resection of the tumor; O (Outcomes): primary outcomes are overall survival, Karnofsky Performance status (KPS) score (for functional outcomes), and effect of increasing age while secondary were miscellaneous outcomes (e.g., death, tumor recurrence); S (Studies): Observational studies and Randomized Controlled Trials.

### 2.3 Data Extraction and Quality Assessment

Two reviewers screened the electronic databases. The EndNote Reference Library version 20.0.1 (Clarivate Analytics, London, UK) was used to extract data, and duplicate articles were removed. Two investigators entered the data extracted from the selected studies into an Excel spreadsheet (Microsoft Corporation, Redmond, WA, USA). The KPS is a simple and quick way to evaluate patients’ performance in daily activities, commonly applied in medical oncology; this score not only helps track the illness's progression but also provides valuable prognostic insights. Variables extracted included KPS, overall survival (OS), patient age, progression‐free survival (PFS), and adverse effects.

Quality assessment was performed using the Newcastle‐Ottawa Scale (NOS) for observational studies and the Cochrane Collaboration Tool for clinical trials. NOS scores of 1–5 were considered high risk for bias, 6–7 were moderate, and scores >7 were considered low risk of bias.

### 2.4 Statistical Analysis

Review Manager (version 5.4.1; Copenhagen: The Nordic Cochrane Centre, The Cochrane Collaboration, 2020) was used for the statistical analyses. The data from studies were pooled using a random‐effects model when heterogeneity was seen. Analysis of results was performed by calculating hazard ratios (HR), odds ratios (ORs), and standard mean differences (SMD), along with their respective 95% confidence intervals (CIs). The chi‐square test assesses any differences between the subgroups. Sensitivity analysis was conducted to assess whether any individual study was driving the results and to explore the reasons for high heterogeneity. As per the *Cochrane Handbook*, the scale for heterogeneity was considered as follows: *I* ^2^ = 25%–60%—moderate, 50%–90%—substantial, 75%–100%—considerable heterogeneity, and *p* < 0.1 indicated significant heterogeneity (Cumpston et al. 2019). A *p*‐value <0.05 was considered significant.

## 3 Results

### 3.1 Literature Search Results

**FIGURE 1:**
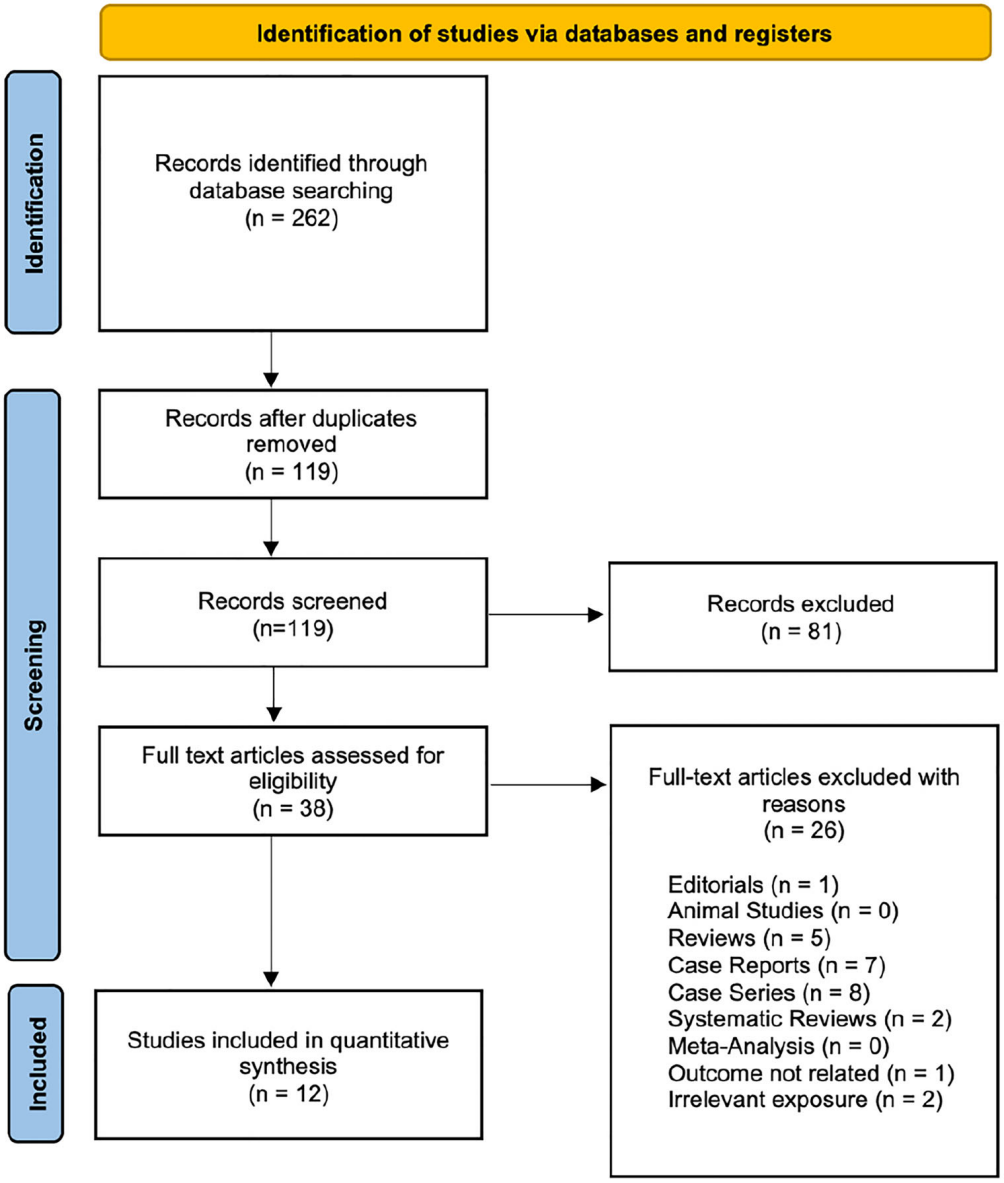
PRISMA flow diagram illustrating the summary of the literature search.

The initial literature review using three electronic databases revealed 262 potential studies. After reviewing the title and abstract, 81 studies were excluded. After duplicate removal, 119 records were screened; 38 full‐text articles were assessed for eligibility, and 26 were excluded. Full‐text exclusions were primarily due to ineligible publication type (e.g., reviews, editorials, case reports/series), non‐clinical studies, or lack of relevant exposure/outcome data. Twelve studies were selected for quantitative analysis. Figure 1 summarizes the results of our literature search.

### 3.2 Study Characteristics

The clinical and demographic details of the selected studies are provided in Table 1 (Di et al. 2022; Roh et al. 2019; Vivas‐Buitrago et al. 2021; Glenn et al. 2018; Lopez‐Rivera et al. 2021; Mampre et al. 2018; Ahmadipour et al. 2019; Pessina et al. 2017; Hirono et al. 2021; Yoo et al. 2021; Shah et al. 2020; Schneider et al. 2019). The total patient population of the 12 cohort studies was 6524. The SMR group included 3317 patients, and the GTR group included 3045 patients. The mean age of the included subjects was 61.2 years.

**TABLE 1:** The baseline table showing the demographic characteristics extracted from the included studies.

Study	Country	Intervention	Total patients (*n*)	Mean age (years)	Female (%)	Outcomes assessed
Di et al. (2022)	USA	SMR GTR	48 54	60.8 61.6	58.3 31.5	KPS, OS, age
Roh et al. (2019)	Korea	SpTR GTR	20 20	62 60	30 35	KPS, OS, age, median PFS, median OS
Vivas‐Buitrago et al. (2021)	USA	SMR	101	59.8	33	KPS, OS
Glenn et al. (2018)	USA	SMR GTR	7 9	56.3 58.1	14.3 22.2	OS, median OS, nonspecified complication, mortality
Lopez‐rivera et al. (2021)	USA	SpTR GTR	2907 2451	72 71	44 43	OS, age
Mampre et al. (2018)	USA	GTR	245	59.8	39	OS
Ahmadipour et al. (2019)	Germany	SpTR GTR	21 234	60.2 62.9	46 38	Age, median OS
Pessina et al. (2017)	Italy	SpTR GTR	21 60	61	37.2	Median OS, nonspecified complication
Schneider et al. (2019)	Germany	GTR	24	68	46	Median OS, median PFS
Hirono et al. (2021)	Japan	SpTR GTR	7 23	56 57	71.4 52.1	Nonspecified complication Local recurrence Distant recurrence
Shah et al. (2020)	USA	GTR	37	65	59.5	Nonspecified complication Mortality
Yoo et al. (2021)	Korea	SpTR GTR	41 194	55.4 55.8	34.1 40.7	Local recurrence Distant recurrence

Abbreviations: GTR, gross total resection; KPS, Karnofsky performance status; OS, overall survival; PFS, progression‐free survival; SMR, supramarginal resection; SpTR, supratotal resection.

### 3.3 Publication Bias and Quality Assessment

All studies had a low risk of bias, except for one which had a moderate risk of bias. Publication bias could not be assessed, as the number of articles in each forest plot was less than 10 (Table 2).

**TABLE 2:** The results of the quality assessment of the included studies.

Study	Selection				Comparability	Outcome			Total Score
	Representation of exposed cohort	Selection of nonexposed cohort	Ascertainment of exposure	Outcome not present at the start of this study		Assessment of OUTCOME	Length of follow‐up	Adequacy of follow‐up	
Pessina et al. (2017)	1	1	1	0	2	1	1	1	8
Glenn et al. (2018)	1	1	1	0	2	1	1	1	8
Mampre et al. (2018)	1	1	1	0	2	1	1	1	8
Ahmadipour et al. (2019)	1	1	1	0	2	1	1	1	8
Roh et al. (2019)	1	1	1	0	2	1	1	1	8
Schneider et al. (2019)	1	1	1	0	2	1	1	1	8
Shah et al. (2020)	1	1	1	0	2	1	1	1	8
(Hirono et al. 2021)	1	1	1	0	2	1	1	1	8
Lopez‐Rivera et al. (2021)	1	1	1	0	2	1	1	1	8
Vivas‐Buitrago et al. (2021)	1	1	1	0	2	1	1	1	8
Yoo et al. (2021)	1	1	1	0	2	1	1	1	8
Di et al. (2022)	1	1	1	0	2	1	1	1	8

*Note*: Risk of bias was assessed using the Newcastle–Ottawa scale (NOS). For selection and outcome items, each criterion is scored 0/1 (criterion not met/met). For the comparability domain, the entry can be 0, 1, or 2 depending on whether the study controlled for none, one, or two prespecified confounders (maximum 2).

### 3.4 Results

**FIGURE 2:**
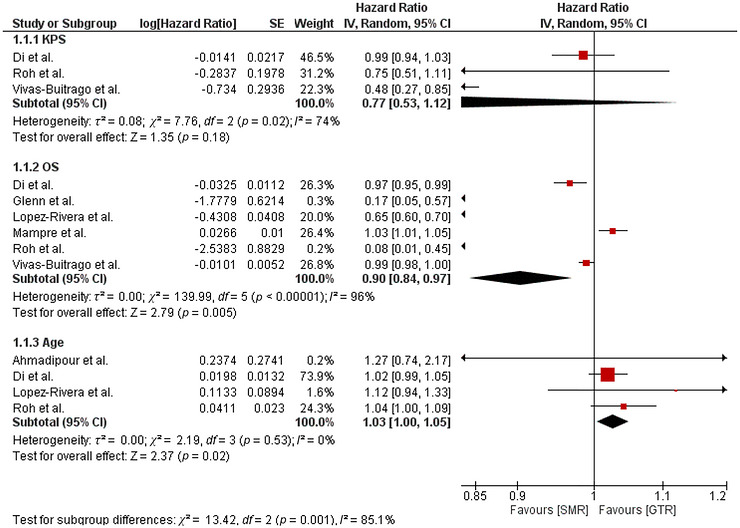
Illustration of the quantitative results of Karnofsky performance status (KPS), overall survival (OS), and age.

**FIGURE 3:**
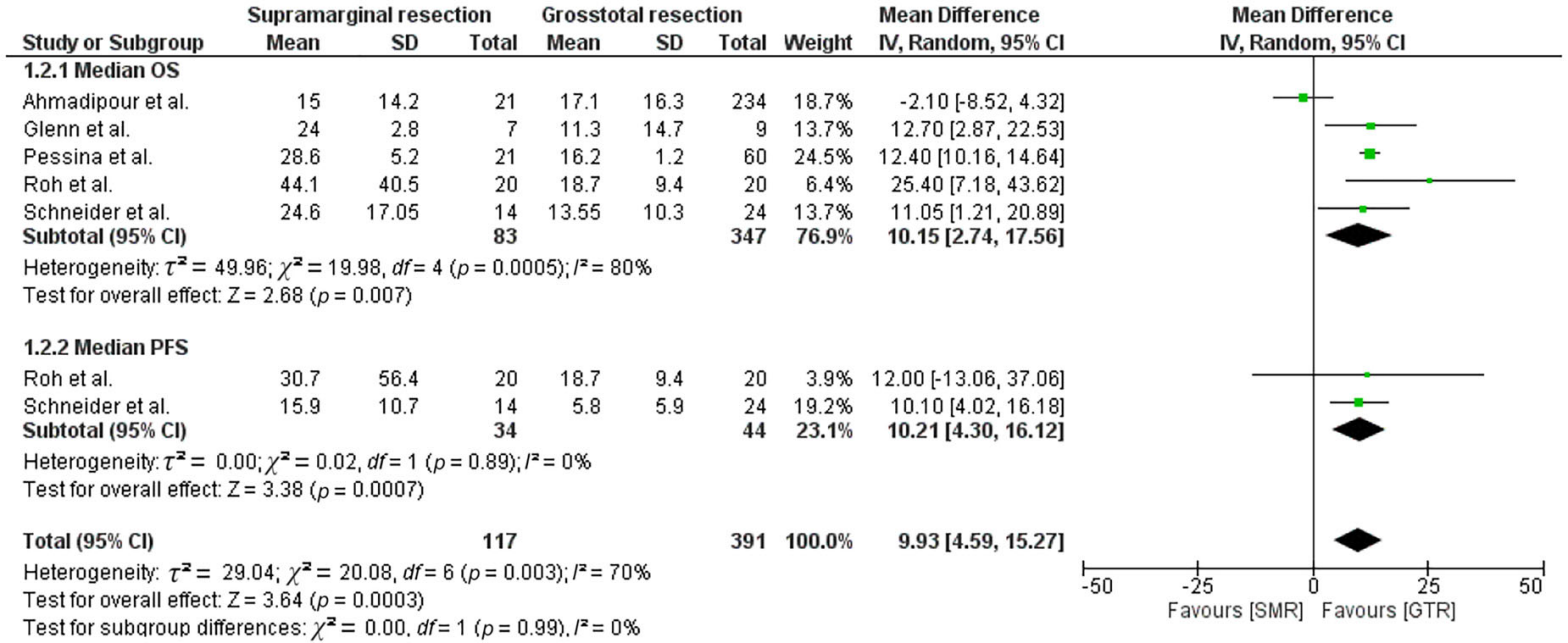
Illustration of the quantitative results for median overall survival (OS) and median progression‐free survival (PFS).

**FIGURE 4:**
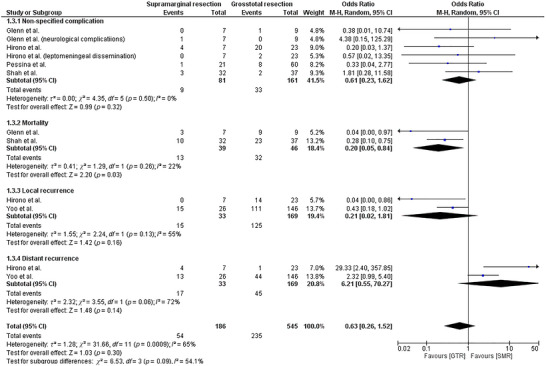
Forest plot illustrating the overall analysis of complications associated with the extent of resection, and subgroup analyses for Non‐specific complications, mortality, local recurrence, and distant recurrence.

Twelve studies were used in this analysis to evaluate the clinical outcomes of GBM patients undergoing surgical resection (Di et al. 2022; Roh et al. 2019; Vivas‐Buitrago et al. 2021; Glenn et al. 2018; Lopez‐Rivera et al. 2021; Mampre et al. 2018; Ahmadipour et al. 2019; Pessina et al. 2017; Hirono et al. 2021; Yoo et al. 2021; Shah et al. 2020; Schneider et al. 2019). Pooled results are given in Figures 2, 3, 4. The following three factors were assessed in the forest plot (Figure 2) to see their effects on the survival outcome of GBM patients: KPS, OS, and age. Figure 2 includes separate pooled models for each factor and demonstrates that effect sizes differ across these factor‐specific analyses (test for subgroup differences: *χ* ^2^ = 13.42, *df* = 2, *p* = 0.001). The forest plot (Figure 3) shows the effect on median OS and median PFS when SMR was compared to GTR in GBM patients. This figure shows that the pooled median OS included five studies (83 SMR/SpTR vs. 347 GTR), while the pooled median PFS included two studies (34 SMR/SpTR vs 44 GTR). Adverse event and recurrence outcomes are summarized in Figure 4 using odds ratios with subgroup and overall pooled estimates (Figure 4).

#### 3.4.1 Karnofsky Performance Status

Three studies were used to analyze the association between lower KPS scores and postoperative survival in GBM patients (Di et al. 2022; Roh et al. 2019; Vivas‐Buitrago et al. 2021). Analysis revealed an insignificant relationship between survival and lower KPS scores (HR = 0.77 [0.53, 1.12]; *p* = 0.18; *I* ^2^ = 74%) (Figure 2). Study weights were 46.5%, 31.2%, and 22.3% (Figure 2). At the study level, the reported HRs ranged from 0.48 (0.27, 0.85) to 0.99 (0.94, 1.03), indicating variability in effect estimates across cohorts.

#### 3.4.2 Overall Survival

Six studies assessed the effects of the extent of resection (supramarginal resection) on OS (Di et al. 2022; Roh et al. 2019; Vivas‐Buitrago et al. 2021; Glenn et al. 2018; Lopez‐Rivera et al. 2021; Mampre et al. 2018). Pooled analysis showed a statistically significant association of OS with SMR in GBM patients (HR = 0.90 [0.84, 0.97]; *p* = 0.005; *I* ^2^ = 96%) (Figure 2). Study weights were uneven (26.8%, 26.4%, 26.3%, 20.0%, 0.3%, and 0.2%), with three studies contributing ∼80% of the pooled estimate (Figure 2). Across individual studies, HRs ranged from 0.08 (0.01, 0.45) to 1.03 (1.01, 1.05), demonstrating wide variation in the magnitude and direction of effects (Figure 2).

The analysis of median OS in GBM patients included five studies (Roh et al. 2019; Ahmadipour et al. 2019; Pessina et al. 2017; Schneider et al. 2019; Al‐Mefty et al. 1996). As compared with the experimental group, GTR had a statistically significant effect on median OS  (SMD = 10.15 [2.74, 17.56]; *p* = 0.007; *I* ^2^ = 80%) (Figure 3).

#### 3.4.3 Age

The effects of age on survival were assessed by four studies (Di et al. 2022; Roh et al. 2019; Lopez‐Rivera et al. 2021; Ahmadipour et al. 2019). A significant association was found between higher age and decreased survival in GBM patients (HR = 1.03 [1.00, 1.05]; *p* = 0.02; *I* ^2^ = 0%).

#### 3.4.4 Progression Free‐Survival

Two studies were used to assess the effects of the experimental and control groups on median PFS (Di et al. 2022; Schneider et al. 2019). Median PFS was significantly improved in the SMR group of GBM patients (SMD = 10.21 [4.30, 16.12]; *p* = 0.0007; *I* ^2^ = 0%). The overall effect of SMR on median PFS and OS in GBM patients was statistically improved, compared to the GTR group (SMD = 9.93 [4.59, 15.27]; *p* = 0.0003; *I* ^2^ = 70%).

#### 3.4.5 Adverse Effects

The forest plot (Figure 4) shows the analysis of the following adverse effects in the two groups of patients: non‐specified complications, mortality, local recurrence, and distant recurrence. Effect estimates are reported as ORs using random‐effects models.

Four studies assessed the risk of non‐specified complications following surgical resection in patients with GBM (Glenn et al. 2018; Pessina et al. 2017; Hirono et al. 2021; Shah et al. 2020). An insignificant difference was noted in the rate of complications between the two groups of patients (SMD = 0.61 [0.23, 1.62]; *p* = 0.32; *I* ^2^ = 0%). This analysis included 81 SMR patients and 161 GTR patients, with 9 versus 33 total events, respectively (Figure 4). No heterogeneity was observed (*τ* ^2^ = 0.00; *χ* ^2^ = 4.35, *df* = 5, *p* = 0.50; *I* ^2^ = 0%).

Two studies reported mortality incidence in GBM (Glenn et al. 2018; Shah et al. 2020). When compared to the GTR, SMR was associated with a significantly lower mortality incidence (SMD = 0.20 [0.05, 0.84]; *p* = 0.03; *I* ^2^ = 22%). This pooled analysis included 39 SMR patients and 46 GTR patients, with 13 versus 32 total events, respectively (Figure 4). Heterogeneity was low (*τ* ^2^ = 0.41; *χ* ^2^ = 1.29, *df* = 1, *p* = 0.26; *I* ^2^ = 22%).

The incidence of local and distant recurrence following surgical resection was assessed by two studies (Hirono et al. 2021; Yoo et al. 2021). Pooled analysis revealed an insignificant difference in the rate of local recurrence in GBM patients (SMD = 0.21 [0.02, 1.81]; *p* = 0.16; *I* ^2^ = 55%). Local recurrence pooled 33 SMR patients and 169 GTR patients, with 15 versus 125 total events, respectively (Figure 4). Heterogeneity was moderate (*τ* ^2^ = 1.55; *χ* ^2^ = 2.24, *df* = 1, p = 0.13; *I* ^2^ = 55%). Similarly, no significant difference was observed in the rate of distant recurrence (SMD = 6.21 [0.55, 70.27]; *p* = 0.14; *I* ^2^ = 72%). Distant recurrence pooled 33 SMR patients and 169 GTR patients, with 17 versus 45 total events, respectively (Figure 4). Heterogeneity was substantial (*τ* ^2^ = 2.32; *χ* ^2^ = 3.55, *df* = 1, *p* = 0.06; I^2^ = 72%), and confidence intervals were wide.

The overall incidence of adverse effects was similar in the two patient groups (SMD = 0.63 [0.26, 1.52]; *p* = 0.30; *I* ^2^ = 65%). Across all adverse‐effect outcomes combined, there were a total of 186 SMR patients and 545 GTR patients, with 54 versus 235 total events, respectively (Figure 4). Overall heterogeneity was moderate (*τ* ^2^ = 1.28; *χ* ^2^ = 31.66, *df* = 11, *p* = 0.0009; I^2^ = 65%). Differences across adverse‐effect subgroups were not statistically significant (test for subgroup differences: *χ* ^2^ = 6.53, *df* = 3, *p* = 0.09; I^2^ = 54.1%) (Figure 4).

## 4 Discussion

The study aimed to assess the impact of factors such as KPS, OS, age, and extent of resection (SMR vs. GTR) on survival outcomes in GBM patients. Specifically, we examined the association between lower KPS scores and postoperative survival, the effect of age on survival, and the impact of SMR versus GTR on median OS and PFS in GBM patients. Additionally, the study explored the adverse effects, including unspecified complications, mortality, local recurrence, and distant recurrence, associated with different surgical resection techniques in GBM patients. The findings aimed to provide comprehensive insights into the factors affecting the prognosis and outcomes of patients with GBM undergoing surgical procedures.

Numerous studies have demonstrated that the extent of removal during surgery is a significant predictor of outcomes in individuals with GBM (D'Amico et al. 2017; Lacroix et al. 2001). It has been consistently observed that patients who undergo more extensive resection experience longer survival times compared to those who receive less aggressive treatment (Sanai et al. 2011). When GTR was achieved, patients experienced a decrease in pseudoprogression and an overall increase in survival time (Park et al. 2016). The median OS doubled compared to when GTR was not achieved (Roh et al. 2017). In our study, analysis of five studies investigating median OS in GBM patients revealed a strong correlation between GTR and improved outcomes compared with the experimental group. There was a statistically significant increase in median OS among GBM patients who underwent GTR (Roh et al. 2019; Glenn et al. 2018; Ahmadipour et al. 2019; Pessina et al. 2017; Schneider et al. 2019).

The literature presented a wide range of evidence supporting the benefits of higher EOR for GBM patients, underscoring its effectiveness in treating this type of cancer (Roh et al. 2019; Vivas‐Buitrago et al. 2021; Glenn et al. 2018; Molinaro et al. 2020; Michaelsen et al. 2013). In patients with GBM undergoing SMR, a critical reference point was identified. A study by Di et al. analyzed 48 SMR patients, with temporal lesions being the most common (39.6%). In contrast, GTR had higher frequencies in temporal (33.3%), frontal (31.5%), and parietal (13%) regions. SMR procedures predominantly utilized awake craniotomy with intraoperative mapping (72.9%) and other mapping techniques. The mean EOR for T1C+ tumor volume was notably high at 97.4%, demonstrating the effectiveness of the SMR approach in achieving thorough tumor removal (Di et al. 2022). Similarly, our analysis of six studies (Di et al. 2022; Roh et al. 2019; Vivas‐Buitrago et al. 2021; Glenn et al. 2018; Lopez‐Rivera et al. 2021; Mampre et al. 2018) examining the influence of SMR on overall survival in patients with GBM revealed a notable, statistically significant correlation, as evidenced by an HR. This discovery implies that individuals who undergo SMR experience a 10% reduction in mortality risk compared to those who do not receive the EOR technique. Shah et al. conducted a substantial comparative study on lobectomy versus oncologic resection (lesionectomy) for newly diagnosed GBM. The study revealed that patients who underwent lobectomy showed significantly better OS and PFS (symptomatic progression) than those who received GTR alone, after adjusting for age, location, and size (Shah et al. 2020). Additionally, SMR is beneficial as it reduces any remaining microscopic disease, thus maximizing the effectiveness of adjuvant treatments (Shah et al. 2020).

In our study, the comparison of mortality incidence between SMR and GTR in GBM, as reported in two studies, showed a significant association (Glenn et al. 2018; Shah et al. 2020). This suggests that patients who underwent SMR had a lower risk of mortality compared to those who received GTR for their GBM treatment, supported by other studies (Di et al. 2022; Michaelsen et al. 2013; Zhao et al. 2019).

Patients with top‐tier Karnofsky scores at the time of tumor diagnosis tend to have better survival rates and quality of life throughout their illness journey (Ferrucci et al. 2007). A study by Chaichana et al. included 80 matched patients who were similar in terms of KPS, age, eloquent involvement, radiation treatment, and temozolomide use. The results showed that surgical resection significantly improved OS compared to needle biopsy without increasing perioperative complications. This finding was consistent even when analyzing a subgroup of 26 patients separately (Chaichana et al. 2011). However, a study by Di et al. included 102 patients, of whom 48 underwent SMR and 54 GTR. The results showed no significant difference in postoperative complication rates or KPS scores between the SMR and GTR groups (Di et al. 2022). Similarly, in our study, we found that the analysis of three studies (Di et al. 2022; Roh et al. 2019; Vivas‐Buitrago et al. 2021). evaluating the effect of lower KPS scores on postoperative survival in GBM patients resulted in a non‐significant relationship. This suggests that lower KPS scores may not significantly affect the postoperative survival of GBM patients. However, previous research has established a clear, strong correlation between higher KPS scores and improved outcomes in this patient population (Liu et al. 2022; Marina et al. 2011).

## 5 Limitations

Our study has some limitations. First, the evidence base included only observational studies, so residual confounding and selection bias may influence the observed associations. Second, relatively few studies contributed to some pooled outcomes, which limits precision and the strength of inference for those factors. Third, several analyses demonstrated substantial heterogeneity across studies, which may limit generalizability. Several included studies predate WHO CNS5, and diagnostic terminology varies across publications (Louis et al. 2021). Finally, postoperative functional measures were not uniformly reported across studies, and the association between KPS and outcomes should be interpreted cautiously; additional prospective work is needed to better define the relationship between KPS and post‐surgical outcomes in GBM (Farooq et al. 2023; Encarnacion‐Santos et al. 2024; Ahmed et al. 2024; Okon et al. 2024; Rybaczek and Chaurasia 2024; Ferini et al. 2024; Gorlia et al. 2008; Lamborn et al. 2004; Curran et al. 1993).

## 6 Conclusion

In this systematic review and meta‐analysis of observational studies, supramarginal resection was associated with improved overall survival compared with gross total resection in patients with glioblastoma. Increasing age was associated with lower survival rates, whereas KPS was not significantly associated with survival. Some of the analyses showed significant heterogeneity, while there was variability in the reporting of adverse events. Hence, this information should be interpreted as associations rather than causal relationships. Further prospective studies with consistent reporting of outcomes and extent of resection, particularly functional outcomes, would help to determine the degree of benefit and which patients would derive the greatest benefit from supramarginal resection.

## Author Contributions

Dipak Chaulagain: concept and design, statistical analysis, first draft of manuscript; Volodymyr Smolanka: study supervision; Andriy Smolanka and Bipin Chaurasia: study supervision, critical revision of the first draft; Oleg Devinyak: study supervision. Kashif Qureshi: manuscript revision in response to first‐round peer‐review comments. Kivanc Yangi: study supervision, critical revision of the first draft. All authors have revised and approved the submitted version.

## Funding

The authors have nothing to report.

## Conflicts of Interest

The authors declare no conflicts of interest.

## Data Availability

Data sharing not applicable to this article as no datasets were generated or analyzed during the current study.
