# Correction to “Efficacy Assessment of Supra‐Marginal Resection Versus Gross Total Resection in Glioblastoma: A Systematic Literature Review and Meta‐Analysis”

**DOI:** 10.1002/brb3.71534

**Published:** 2026-06-04

D. Chaulagain, V. Smolanka, A. Smolanka, et al. 2026. “Efficacy Assessment of Supramarginal Resection Versus Gross Total Resection in Glioblastoma: A Systematic Literature Review and Meta‐Analysis” Brain and Behavior 16, no. 4: e71424. https://doi.org/10.1002/brb3.71424

In the published article, the author's name “Oleg Devnyak” was misspelled. The correct author's name is “Oleg Devinyak”. In addition, their affiliation should be corrected from “Pharmacology Department, Uzhhorod National University, Uzhhorod, Ukraine” to “Department of Pharmaceutical Sciences, Uzhhorod National University, Uzhhorod, Ukraine.”

*The online version of this article has been corrected accordingly*.

We apologize for these errors.

